# Text Message Reminders and Unconditional Monetary Incentives to Improve Measles Vaccination in Western Kenya: Study Protocol for the Mobile and Scalable Innovations for Measles Immunization Randomized Controlled Trial

**DOI:** 10.2196/13221

**Published:** 2019-07-09

**Authors:** Dustin G Gibson, E Wangeci Kagucia, Joyce Were, David Obor, Kyla Hayford, Benard Ochieng

**Affiliations:** 1 Department of International Health Johns Hopkins Bloomberg School of Public Health Baltimore, MD United States; 2 International Vaccine Access Center Department of International Health Johns Hopkins Bloomberg School of Public Health Baltimore, MD United States; 3 Center for Global Health Research Kenya Medical Research Institute Kisian Kenya

**Keywords:** measles vaccine, text messaging, Kenya, vaccination coverage

## Abstract

**Background:**

Globally, 21 million children do not receive the measles vaccine each year. With high levels of mobile phone access and ownership, opportunities exist to leverage mobile health technologies to generate demand for immunization.

**Objective:**

The aim of the Mobile and Scalable Innovations for Measles Immunization trial is to determine if text message (short message service, SMS) reminders, either with or without mobile phone–based incentives, can improve measles immunization coverage and timeliness in rural western Kenya.

**Methods:**

This is a 3-arm, parallel, randomized controlled trial (RCT). Using simple randomization, caregivers in Siaya County, Kenya, will be randomized and evenly allocated to 1 of 3 study arms: (1) control, (2) SMS reminders only, and (3) SMS reminders plus a 150 Kenyan Shilling (KES) incentive. Participants assigned to the SMS group will be sent SMS reminders 3 days before and on the day before the measles immunization visit scheduled for when the child is 9 months of age. Participants in the incentive arm will, in addition to SMS reminders as above, be sent an unconditional 150 KES mobile-money incentive to their mobile phone 3 days before the child becomes 9 months of age. Children will be followed up to the age of 12 months to assess the primary outcome, a measles vaccination by 10 months of age. Log-binomial regressions will be used to calculate relative risks.

**Results:**

Enrollment was completed in March 2017. We enrolled 537 caregivers and their infants into the following groups: control (n=179), SMS reminders only (n=179), and SMS reminders plus 150 KES (n=179). Results will be made publicly available in 2020.

**Conclusions:**

Few RCTs have examined the effect of text message reminders to improve measles immunization coverage. This is the first study to assess the effect of SMS reminders with and without unconditionally provided mobile-money incentives to improve measles immunization coverage.

**Trial Registration:**

ClinicalTrials.gov NCT02904642; https://clinicaltrials.gov/ct2/show/NCT02904642 (Archived by WebCite® at http://www.webcitation.org/78r7AzD2X).

**International Registered Report Identifier (IRRID):**

RR1-10.2196/13221

## Introduction

### Background

In 2017, 20.8 million age-eligible children worldwide did not receive measles-containing vaccine [1]. Measles is a highly contagious virus, which is evident from measles outbreaks occurring in populations comprising as little as 10% susceptible, or immunologically unprotected, individuals [2]. Given that every year immunization programs are estimated to save over 3 million lives globally [3], innovative interventions that are easily scaled and that target hard-to-reach and underimmunized populations are needed.

Mobile phone technologies and tools, broadly referred to as mobile health, have been used successfully for a variety of public health problems [4,5] and to strengthen and improve health systems [6,7]. The Classification of Digital Health Interventions, released by the World Health Organization in 2018, categorizes digital health interventions by the health systems challenges they address [8]. As mobile phone ownership and access levels continue to rise in lower income countries [9], digital health solutions that address demand-side deficiencies in immunization services become more realistic.

Short message service (SMS), or *text message*, reminders and incentives are 2 of the more common interventions that have been used to generate demand for immunization services [10,11]. Text message reminders have been shown to modestly improve immunization uptake in studies in the United States and other high-income countries [12]; although their evidence to improve uptake in low- and middle-income countries (LMICs) is limited. [13].

With regard to incentives, at least 4 studies have evaluated the impact of small monetary and nonmonetary incentives, such as coupons and food, on vaccination in LMICs. Studies in India [14], Kenya [15-17], and Pakistan [18] found that monetary and nonmonetary incentives have the potential to improve vaccination coverage and also suggest that incentive-based interventions have the potential to affect hard-to-reach populations. Moreover, all of the identified studies provided a conditional incentive, where the caregiver was only given the incentive if the child was vaccinated. To our knowledge, no studies have provided unconditional incentives for vaccination.

This study builds on the success of the recently completed Mobile Solutions for Immunization (M-SIMU) cluster randomized controlled trial (RCT). The M-SIMU trial found that providing a 200 Kenyan Shilling (KES) mobile-money incentive, or approximately US $2.50 at the time of the study, conditionally to caregivers who vaccinated their child within 2 weeks of the Expanded Program on Immunizations (EPI) due date significantly improved the proportion of fully immunized children and measles vaccination coverage measured at 12 months [15,19]. A conditional incentive approach may be difficult to scale because it requires staff at the clinic to document immunization and to calculate whether the immunization was received in time. Additionally, unconditional incentives may be more effective at increasing the intended health outcome if the transferred money is used to offset incurred travel costs, rather than receiving the money after visiting the health facility, as in a conditional approach.

### Objective

To counter these logistical and implementation challenges and to facilitate their scalability, the Mobile and Scalable Innovations for Measles Immunizations (M-SIMI) RCT seeks to evaluate the impact of providing rural Kenyan caregivers SMS reminders and unconditional monetary incentives on measles immunization coverage at 10 months of age.

## Methods

### Study Design

**Figure 1:**
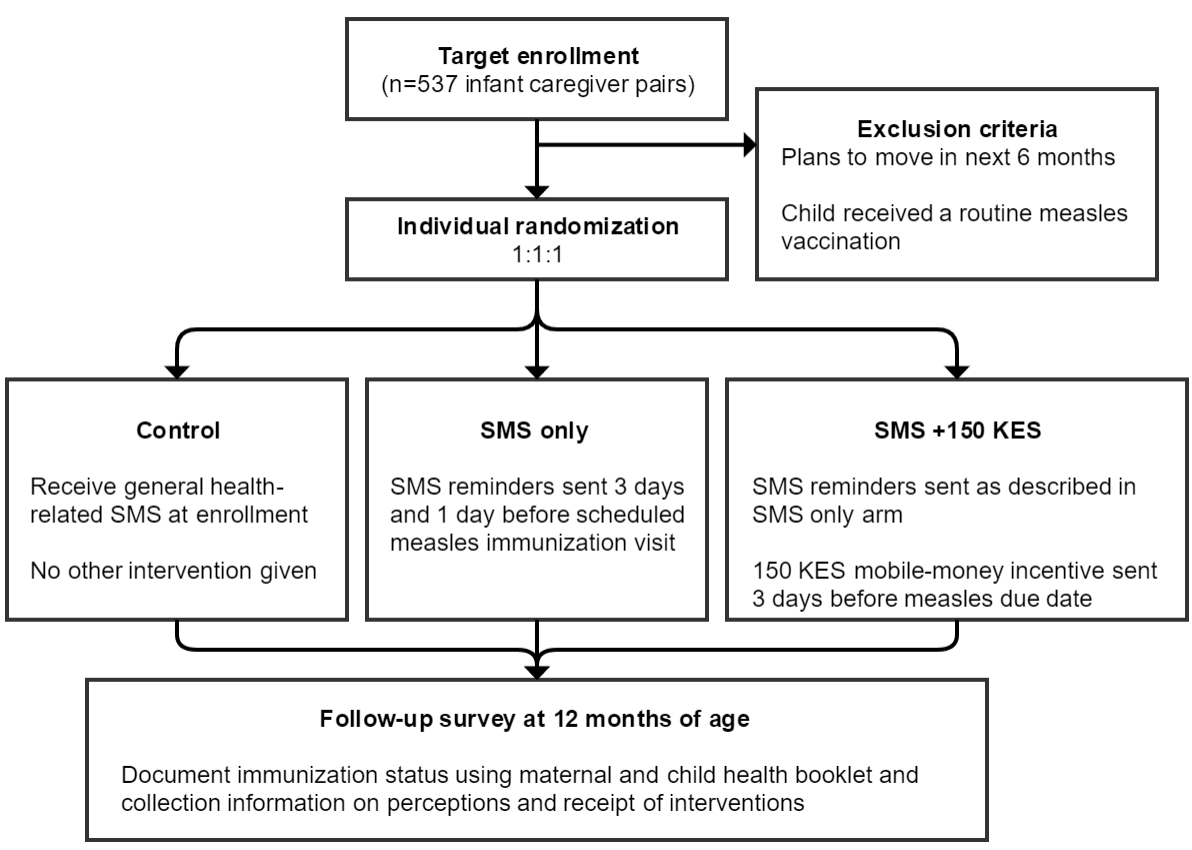
Consolidated Standards of Reporting Trials diagram of study design. SMS: short message service; KES: Kenyan Shilling.

The M-SIMI study is an individually randomized, parallel, controlled trial (Figure 1). Caregivers will be randomized into 1 of 3 study arms using a 1:1:1 allocation ratio. The study arms include (1) control, (2) text message reminders only (SMS only), and (3) text message reminders plus a 150 KES incentive (SMS plus 150 KES); where 100 KES=US $1 as of June 2016. Participants randomized to the intervention arms will be sent 2 SMS reminders—3 days and 1 day before measles immunization visits scheduled at 9 months of age. Incentives will be unconditionally sent to the participant’s mobile phone 3 days before the measles vaccination due date at 9 months of age. The trial is registered with ClinicalTrials.gov (NCT02904642; September 2016).

### Setting and Participants

The M-SIMI study will take place within the boundaries of the Kenya Medical Research Institute (KEMRI) and the Centers for Disease Control and Prevention (CDC) Health and Demographic Surveillance System (HDSS) in Siaya County, Kenya. The rural study site has high levels of malaria, tuberculosis, and HIV transmission [20]; the majority of residents are subsistence farmers; and approximately 50% of caregivers own a mobile phone [21]. Data from the control arm of our previously conducted RCT found that 98% and 51% of children received pentavalent and measles vaccination by the age of 12 months, respectively [15].

The HDSS has served as a platform for numerous studies, including the M-SIMU trial, which provided text message reminders and conditional mobile-money incentives for pentavalent and measles vaccinations [15]. Potential caregivers and their children will be identified by community health workers (CHWs). CHWs are a component of Kenya’s national community health strategy and typically have a catchment area of 1 village, where the CHW visits each household at least once a month to provide minimal health services and education. Community interviewers (CIs) hired by the project will approach CHW-identified caregivers to describe the study and screen caregivers for their eligibility on the following criteria (see Textbox 1).

Caregivers will be enrolled into the study independent of mobile phone ownership. Caregivers only need to have access to a mobile phone, where access will be defined by the participant. If a caregiver cannot identify an accessible mobile phone, CIs will relay text message reminders and incentives to the participant. Caregivers will neither be provided a mobile phone nor airtime.

**The Mobile and Scalable Innovations for Measles Immunizations screening criteria.**

Inclusion criteria:

- Caregiver of infant aged 6 to 8 months at the time of enrollment
- Self-reported resident of one of the study villages
- Willing to sign informed consent for the study

Exclusion criteria:

- Child of enrolled caregiver has already received 1 dose of measles vaccine, not including any supplemental measles vaccines
- Caregiver plans to move away in the next 6 months

### Procedures

#### Enrollment

During their monthly visit, CHWs will identify households with children aged 6 to 8 months. CHWs will approach caregivers of age-eligible children, provide a brief explanation of the study, and answer any questions the caregiver might have. The CHWs will call the field supervisor to notify the study team that they have identified a child aged 6 to 8 months. The field supervisor will then assign a CI to visit the child’s compound to explain the trial and screen the caregiver for the eligibility criteria as described above.

Eligible caregivers will be required to provide written informed consent to the CI. Upon obtaining written informed consent, the CI will send an enrollment SMS to the RapidSMS server, a free and open-source platform. The enrollment SMS is structured and requires the CI to enter the study identification number, the phone number that can be used to receive text message reminders, the child’s date of birth, the preferred language to receive reminders, the child’s first and last name, and the study arm. If the CI sends an incorrectly formatted SMS, he or she will receive an error SMS, which asks the CI to correct the SMS. The RapidSMS server will then send a personalized text message to the newly enrolled caregiver that welcomes him or her to the study (Table 1). The CI will confirm receipt of the enrollment SMS.

#### Control Arm

Aside from the welcome text message received at the enrollment visit, no additional text messages or incentives will be sent to caregivers randomized to the control arm.

#### Intervention Arms

The interventions, text message reminders, and unconditional incentives are designed to motivate caregivers and increase demand for measles vaccination. At enrollment, participants who were randomized to an intervention arm will be told to expect 2 text message reminders for measles vaccine; first, at 3 days before and, second, on the day before the scheduled visit for measles vaccination at 9 months of age. At most, enrolled caregivers will receive 3 text messages (2 for the measles vaccine and 1 for welcoming the caregiver to the study). Text messages will be sent in English, Kiswahili, or Dholuo language, according to the caregiver’s preference.

Caregivers randomized to the incentive study arm will, in addition to being told about SMS reminders as described above, be informed of the measles vaccine due date and that the project will send a 150 KES incentive 3 days before the child turns 9 months (ie, sent on the same day as the first SMS reminder). Caregivers will receive this one-time mobile-money incentive unconditionally. The incentive is intended to subsidize the cost of transportation to the health facility. The transaction costs associated with mobile-money transactions will be borne by the study such that caregivers will receive the full 150 KES. The incentive amount was informed by results of the M-SIMU trial [15].

### Randomization

Caregivers will be evenly randomized (1:1:1) to 1 of 3 study arms. A computer will randomly allocate the study arm to 537 unique study identification numbers. The study identification numbers will be divided into 5 groups (for each of the 5 CIs who will enroll caregivers) and labeled A to E. Each group will contain 109 study identification numbers, labeled 1 to 109. For example, group A will have study IDs labeled 001A through 109A, group B will have study IDs labeled 001B through 109B, etc. The allocation code will be saved on the data manager’s computer. The allocation will be printed on a small card and placed in an opaque envelope by the data analyst. The envelopes will be sealed and stamped to ensure that they cannot be tampered with. Biweekly, the study coordinator will deliver the sealed envelopes to the field supervisor who will then distribute them to the CIs.

CHWs will identify eligible caregiver-infant pairs and place a phone call to the field supervisor to provide information on the location of the child. The field supervisor will notify CIs who will then approach caregivers of age-eligible children to conduct a screening for further eligibility requirements and to obtain informed consent. If the caregiver is eligible for the study and decides to participate, the CI will open the envelope containing the study arm and read the appropriate informed consent form to the caregiver.

**Table 1:** Content of text messages sent to caregivers’ mobile phones.

Message type	Message timing	Arm 1: control	Arm 2: SMS^a^ only	Arm 3: SMS+150 Kenyan Shilling
Enrollment message	Enrollment	Thank you for enrolling Baby <BABY’S FIRST NAME> in M-SIMI^b^ study. The greatest wealth is health. This study is sponsored by KEMRI^c^.	Thank you for enrolling your child in the KEMRI M-SIMI study. You will get SMS reminders for Baby <BABY’S FIRST NAME>’s measles vaccination. The greatest wealth is health.	Thank you for enrolling your child in the KEMRI M-SIMI study. You will get SMS reminders for Baby <BABY’S FIRST NAME>’s measles vaccination. The greatest wealth is health.
3 day reminder message	Date of birth + 9 months – 3 days	No message	Tell Mama <BABY’S FIRST NAME> that Measles vaccine is due this week. Most Gem babies get vaccinated, be one of them!	Tell Mama <BABY’S FIRST NAME> that Measles vaccine is due this week. We are sending 150ksh to assist with travel. Most Gem babies get vaccinated, be one of them!
1 day reminder message	Date of birth + 9 months – 1 day	No message	Tell Mama <Baby Fname> that Measles vaccine is due this week. Go to the clinic if you haven’t already. Vaccines save Kenyan babies lives.	Tell Mama <Baby Fname> that Measles vaccine is due this week. Go to the clinic if you haven’t already. Vaccines save Kenyan babies lives.

^a^SMS: short message service.

^b^M-SIMI: Mobile and Scalable Innovations for Measles Immunizations.

^c^KEMRI: Kenya Medical Research Institute.

Due to the nature of the intervention, participants will not be blinded to the study arm. Additionally, because this trial is conducted to mimic circumstances of what a scaled reminder or travel subsidy program would look like, participants will be told which study arm they have been assigned to at enrollment. As an example, in a scaled program, caregivers would know that they would receive an incentive when their child reached the age of 9 months. Trained interviewers will not be blinded to study arm allocation because of the nature of the trial.

### Data Collection

The enrollment survey, which will collect baseline health and sociodemographic data about the child, caregiver, and household, will be administered by CIs using a mobile phone equipped with Open Data Kit software. Specifically, we will collect information on the caregiver’s phone ownership and usage and the caregiver’s demographics including age, education, marital status, and literacy level, household assets, number and type of livestock owned, water source, type of cooking fuel used, and current occupation. When children reach 12 months of age, a CI will conduct a household visit to document measles immunization status. Caregivers will be asked to present their maternal and child health (MCH) booklet so that CIs can record a written immunization history of Bacillus Calmette–Guérin, pentavalent, polio, and measles vaccines. If the MCH booklet is unavailable, CIs will collect a verbal report of the child’s immunizations. If the child is found to not be up to date with all vaccinations, the CI will refer the caregiver and infant to the nearest clinic, but the study will not pay for transport or health care costs. In addition to the immunization history, the follow-up survey will ask questions surrounding text message reminders and incentives, as applicable. Costs of the interventions (eg, SMS, mobile-money incentive and staff time) will be collected throughout the study for future cost-effective analyses.

Data will be stored in a secure database at KEMRI Center for Global Health Research (KEMRI/CGHR) in Kisian, Kenya. Photo identification and key cards are required to enter the data storage area. Only a limited number of authorized staff will have access to the data. Data will be deidentified for analysis and will only be shared with investigators of the study protocol. Caregivers will be assigned a study ID number in line with current HDSS operating procedures. Linkages with HDSS data will be made so that the CHW enrollment approach can be compared with existing HDSS population estimates.

### Outcomes

The primary outcome is the proportion of children who received measles vaccination by 10 months of age. Measles vaccination at 12 months is a secondary outcome. Data for primary and secondary immunization outcomes will come from written immunization records found on the child’s MCH booklet at household follow-up visits. If dates in MCH booklet are illegible, missing, or vaccination history was provided verbally, the health facility immunization registries will be searched to identify vaccines given. Data from our previous study conducted in the same setting indicated that over 95% of caregivers had an MCH booklet at 12 months of age [15].

### Data Analysis

The analysis and reporting of results will be conducted in accordance with the Consolidated Standards of Reporting Trials [22]. A blinded statistician will conduct analyses for primary and secondary outcomes. The primary analyses will be conducted with intention-to-treat principles. The primary outcome, measles immunization at 10 months of age, will be defined as a binary variable. Log-binomial regressions to calculate risk ratios for achieving the primary outcome will be calculated for the intervention arms as compared with the control arm. As a secondary analysis of the primary outcome, time-to-immunization curves will be constructed using the Kaplan-Meier method, and study arms will be compared using the Cox model. The 25th, 50th, and 75th percentiles for time to measles immunization will also be calculated by the study arm. Effect estimates will be presented in whole and stratified on mobile phone ownership and clinic proximity. Mobile phone ownership will be defined as a binary variable (*owns a mobile phone* or *doesn’t own a mobile phone*). Additional stratified estimates will be presented based on calculated risk factors for not receiving measles vaccine in control arm infants. Adjusted analyses will be conducted if our randomization produced imbalanced sociodemographic characteristics by the study arm. A per-protocol analysis of primary and secondary outcomes will also be conducted. Per-protocol delivery of the interventions will be defined as caregivers who were sent 2 text message reminders for measles vaccination in the 2 intervention arms and as caregivers who were sent an incentive in the third study arm.

Socioeconomic quintile scores will be calculated using a multiple correspondence analysis of household assets, livestock owned, water source, type of cooking fuel used, and occupation [23]. In villages where HDSS and CHWs are active, we will conduct a sensitivity and specificity analysis of our CHW approach to identify age-eligible children. The HDSS database, which relies on village reporters to enumerate the population, will be used as the gold standard. We will compare the costs of a CHW versus HDSS approach of identifying eligible children. An alpha of .05 will be assumed for all statistical tests of significance.

### Sample Size

The primary objective is to assess the effect of the interventions on their ability to improve measles vaccination coverage by an absolute difference of 15 percentage points at 10 months of age as compared with the control arm (eg, 70% measles coverage in the control arm and 85% coverage in an intervention arm). This effect size was selected because it represents a meaningful public health impact and could motivate decision makers to adopt this intervention. Data for the sample size calculation come from the M-SIMU trial; where we found a measles vaccination coverage of 70% at 10 months of age in control arm children [15]. Assuming a 70% outcome in the control group, an absolute 15% difference in the primary outcome between control and intervention arms, a type 1 error (alpha) of .05 and a power (1-beta) of 0.80, we calculated that 134 caregivers are needed in each study arm. Adjusting our sample size for a 25% loss to follow-up (death, outmigration, verbal report of measles vaccination at 10 months of age, and other reasons), our adjusted sample size is 179 participants per study arm, yielding a total sample size of 537 caregivers across the 3 study arms.

### Ethical Considerations

This study protocol received ethical clearance from the Scientific Steering Committee, the KEMRI Scientific and Ethics Review Unit (SERU; KEMRI/SERU/CGHR/003/3311; Multimedia Appendix 1), and the Johns Hopkins University Bloomberg School of Public Health (deferred ethical clearance to KEMRI-ERC). All participants will provide written informed consent. If a participant is illiterate, consent forms will be administered and signed in front of an impartial witness. At the 12-month household visit, study staff will refer the caregivers of unvaccinated children to the nearest health facility that provides measles vaccination.

## Results

M-SIMI completed enrollment in March of 2017. We met our sample size requirement and enrolled 537 caregivers and their infants into the following groups: control (n=179), SMS reminders only (n=179), and SMS reminders plus 150 KES (n=179). Results will be made publicly available in 2020.

## Discussion

### Differences Between Previous Studies

This proposed study differs from our previous cluster RCT, the M-SIMU study, in several ways [15,19]. First, M-SIMU enrolled infants younger than 5 weeks and provided reminders and incentives for the pentavalent vaccine series at 6, 10, and 14 weeks of age and for measles vaccine at 9 months of age. The significant effect of incentives on measles coverage may be due to caregivers previously receiving travel subsidies and reminders for the 3 pentavalent doses. Contrastingly, the M-SIMI study seeks to enroll caregivers of children aged 6 to 8 months and to provide reminders and unconditional travel subsidies only for measles vaccination.

A second difference, and perhaps a more important one, is that M-SIMU provided conditional incentives for vaccination, where the incentive was only given if the caregiver brought their child for vaccination within 2 weeks of the EPI due date. M-SIMU–employed health facility recorders were stationed at each clinic to confirm a timely vaccination. With busy clinic workers, this conditional approach is difficult to scale because it requires staff at the clinic to document immunization and to calculate whether the immunization was received in time. To counter these logistical and implementation challenges, the M-SIMI trial will provide an incentive unconditionally, that is, the caregiver receives an incentive 3 days before the child’s 9-month birthday and independent of whether the child has been vaccinated for measles.

A third difference between M-SIMU and M-SIMI is in the enrollment strategy. Where the M-SIMU trial employed KEMRI HDSS village reporters to identify births and deaths, this proposed study seeks to leverage CHWs, a component of Kenya’s national community health strategy, to identify and enroll children into a travel subsidy scheme. Village reporters are a unique component of the HDSS, making the external generalizability of our results somewhat limited, whereas numerous countries have national CHWs.

### Limitations

This study has several potential limitations. First, not all caregivers will provide written immunization records at the 12-month follow-up visit. Evidence from other settings implies that they may under or overestimate actual vaccination coverage [24-26]. It is likely that social desirability bias may lead those who received an incentive in this study to verbally report vaccination. At the same time, this bias may be minimal as the M-SIMU trial found that over 95% of caregivers had an MCH booklet at 12 months of age [15]. An additional limitation of this study is that the CIs who will collect measles vaccination status at the follow-up visit will also be responsible for assigning the study arm at enrollment (ie, not blinded to study arm allocation). To minimize the risk of outcome ascertainment bias, the follow-up questionnaire will be structured to collect vaccination data before any questions that identified the study arm allocation. For CIs to be biased when ascertaining vaccination status, they would have had to rely on their memory of the participant’s study arm allocation during the enrollment visit, which was conducted 4 to 6 months before the follow-up visit.

We hypothesize that the interventions will improve measles vaccination coverage and that they will be effective in many of the traditionally hard-to-reach populations. By designing this study with scalability in mind, the results of this trial will hopefully approximate the effects of national or subnational programs.

## Appendix

Multimedia Appendix 1 Center Scientific Committee review comments.

## Abbreviations

CDC: Centers for Disease Control and Prevention
CHW: community health worker
CI: community interviewer
EPI: Expanded Program on Immunization
HDSS: Health and Demographic Surveillance System
KEMRI: Kenya Medical Research Institute
KES: Kenyan Shilling
LMICs: low- and middle-income countries
MCH: maternal and child health
M-SIMI: Mobile and Scalable Innovations for Measles Immunization
M-SIMU: Mobile Solutions for Immunization
RCT: randomized controlled trial
SMS: short message service
