# Supplementary material for: Text Message Reminders and Unconditional Monetary Incentives to Improve Measles Vaccination in Western Kenya: Study Protocol for the Mobile and Scalable Innovations for Measles Immunization Randomized Controlled Trial
Source: JMIR Res Protoc. 2019 Jul 9;8(7):e13221. doi: 10.2196/13221 (PMC6647752; doi:10.2196/13221)
Supplement: Multimedia Appendix 1 [file resprot_v8i7e13221_app1.pdf]

Protocol review report from Caroline Makokha

Protocol title: Randomized Controlled Trial of the Impact of Mobile Phone Delivered Reminders and Unconditional Travel Subsidies on Measles Vaccination in Western Kenya: The Mobile and Scalable Innovations for Measles Immunization (M-SIMI) Trial

This is an important study that has the potential for scale up countrywide if successful. However, there are a few comments that need to be addressed:

1. The rationale for unconditional travel subsidy is not clear
2. Important references are missing e.g. Kenya's community strategy
3. Contradicting statements in the justification for the study vs the study procedures for the interventions arm
4. Your definition for residence as an inclusion criteria is lacking
5. You mention in the introduction section that the travel subsidy will help **poor** mothers offset costs incurred in taking the child for vaccination and that is not followed by information on the poverty/ wealth index and socioeconomic activities of the study are under the study site section.
6. Under the study site, you mention that the study site has high levels of HIV, TB, and malaria; high is relative, what is high for this important health indicators.
7. It's not clear how the HDSS identified children will be involved in this study-secondary objective 4
8. The other minor comments and editing errors are in the document track changes.
